# Linkage Among Cognition, Emotion and Behavior: An Interdisciplinary Perspective

**DOI:** 10.3390/brainsci15090962

**Published:** 2025-09-04

**Authors:** Carmelo Mario Vicario, Gabriella Martino

**Affiliations:** 1 Department of Cognitive, Psychological, Educational and Cultural Studies, University of Messina, 98122 Messina, Italy; 2 Department of Clinical and Experimental Medicine, University of Messina, 98122 Messina, Italy; gabriella.martino@unime.it

The Special Issue “Linkage among Cognition, Emotion and Behavior” offers a comprehensive exploration of the dynamic relationships between the emotional, cognitive, and behavioral domains. The contributions in this collection collectively highlight the crucial role of emotional and cognitive processes in shaping human behavior across clinical, educational, and social contexts.

Several of the contributions explore the biological underpinnings of emotional and cognitive processes. Vega-Rosas et al. (Contribution 1) report that higher levels of enriched environments are associated with increased serum brain-derived neurotrophic factor (BDNF) levels in patients with Major Depressive Disorder, reinforcing the role of environmental stimulation in promoting neuroplasticity. Complementarily, prior research has pointed out other methods of effectively inducing neuroplasticity for clinical/therapeutic purposes, such as non-invasive brain stimulation [1,2,3,4,5,6,7,8,9,10] and exposure therapy [11,12,13,14].

In the domain of emotional and cognitive flexibility, Feng et al. (Contribution 2) highlight that “freely moving mind wandering” (FMMW) supports post-incubation creative performance, emphasizing the importance of dynamic internal cognition for creativity. Relatedly, Binsaeed et al. (Contribution 3) show that emotional and cultural intelligence are critical predictors of innovative work behavior in the healthcare sector, further illustrating how emotional competencies can scaffold cognitive and behavioral adaptability.

Emotion regulation and empathy are also central themes in this Special Issue. Imajo et al. (Contribution 4) demonstrate that allowing individuals to choose their own emotion regulation strategies enhances stress reduction, while Lee et al. (Contribution 5) show that activating facial muscles enhances empathic pain, in line with the facial feedback hypothesis. These findings resonate with theoretical discussions on the neurobiological roots of moral behavior [15,16,17], suggesting that emotional self-regulation and embodied emotional experiences—which can be altered by several factors, including addiction [18,19], and be related to individual variables [20,21]—play a key role in ethical and prosocial behaviors.

Moral cognition is specifically addressed by Lin et al., (Contribution 6) who apply drift–diffusion modeling to show that moral evaluations are primarily driven by emotional intuitions, supporting the social intuitionist model of morality proposed by previous theoretical and experimental investigations in this field [22,23]. The strong emotional basis of moral reasoning is an important theme for understanding not only everyday ethical judgments but also clinical alterations in emotional–moral processing [24,25].

Educational and developmental contexts are represented by Taniguchi et al., (Contribution 7) who found that metacognitive skills and personality traits predict mental well-being in students enrolled in therapy programs. These findings align with emerging educational tools, such as virtual reality applications, which are being explored for their potential to foster cognitive and emotional skills [26,27,28,29,30,31,32,33].

Several papers in this Special Issue also address the clinical and psychopathological implications of emotional–cognitive linkages. Grażka and Strzelecki (Contribution 8) provide a systematic review highlighting how early maladaptive schemas relate to suicidality, while Tortora et al. (Contribution 9) emphasize the serotonergic modulation of fear learning and memory, consolidating evidence that neurotransmitter systems underlie critical emotional–cognitive behaviors.

Attentional mechanisms and emotion-driven salience are addressed by Li et al., (Contribution 10) who reveal that the prioritized detection of fearful eyes during attentional blink is not automatic, highlighting the limits of rapid emotional processing under cognitive load. Related investigations into cognitive dissonance and memory in aging [34,35,36,37,38,39] further stress the dynamic adjustments between cognition and emotional preferences across the human lifespan.

In summary, this Special Issue offers a heterogeneous and integrative set of studies that expand our understanding of the intricate relationships between cognition, emotion, and behavior. It also connects with broader fields such as clinical neuroscience, educational innovation, and moral psychology, opening avenues for future research and interdisciplinary collaboration.
